# Mechanical Response and Failure Mechanisms of Cable-Bolt Reinforcement Under Impact Loading

**DOI:** 10.3390/ma19214757

**Published:** 2026-11-09

**Authors:** Jianhang Chen, Yang Yang, Cunwen Wang, Chaohong Shi, Krzysztof Skrzypkowski, Krzysztof Zagórski

**Affiliations:** 1 Shandong Energy Group, Jinan 250101, China; 2 School of Energy and Mining Engineering, China University of Mining and Technology (Beijing), Beijing 100083, China; 3 Faculty of Civil Engineering and Resource Management, AGH University of Krakow, 30-059 Krakow, Poland; 4 Faculty of Mechanical Engineering and Robotics, AGH University of Krakow, 30-059 Krakow, Poland

**Keywords:** rock bursts, anchorage grout, steel materials, impact loading, cable material failure

## Abstract

With mining depth increasing, rock burst disasters are likely to occur and induce impact loading on the cable-bolt reinforcement. Based on this fact, this paper conducted a literature review on the mechanical response and failure mechanisms of cable-bolt reinforcement under impact loading. Two different experimental testing methods were summarised, including the horizontal high-speed impact-loading test and vertical drop-hammer impact-loading test. The advantages and disadvantages of these two experimental test methods were summarised. Compared with the horizontal high-speed impact-loading test, when the vertical drop-hammer impact-loading test was used, the impact energy can be adjusted conveniently. Additionally, the maximum impact velocity can be increased to several times. The influence of different parameters on the mechanical response of the cable-bolt reinforcement was reviewed, including the cable-bolt structure, impact energy and pretension force. Considering that the impact energy may be applied on the cable-bolt reinforcement along the transverse direction, the shear load-bearing behaviour of the cable-bolt reinforcement was also reviewed. Following this, when the impact loading occurred, the cable-bolt reinforcement failure modes were summarised, including the cable-bolt rupture, cable-bolt slippage and locking-nut slippage. This research is beneficial for researchers to better understand the mechanical response and failure mechanisms of the cable-bolt reinforcement subjected to impact loading.

## 1. Introduction

In China, the coal resource distribution is largely different. In fact, the majority of coal resources are located in deep rock strata. Restricted by the deep coal resource distribution state, in China, a large number of coal mines belong to deep coal mining. Under the highly intensified coal mining, the mining depth in China is continuously increasing.

Under the deep mining condition, there are serious issues including high in situ stresses, high ground temperature, high osmotic pressure and intensified mining influence [1]. Under the multiple and complicated influence of in situ stresses, the bursting liability of coal and rock is changing from weak to strong. When the bursting liability of coal and rock is strong, under the mining influence, dynamic disasters are likely to occur, such as rock bursts. This seriously threatens the safety of underground operators in coal mines. Also, this directly restricts the high production and efficiency of coal mines.

Previous research indicates that more than 90% of rock bursts occur in roadways [2]. Additionally, the most severe failure induced by rock bursts occurs in roadways [3]. Therefore, anti-impact support and reinforcement are significant components in ground control of deep roadways in mines [4].

In ground control of deep roadways, cable bolts are significant components in active reinforcement in coal mines [5,6]. Cable bolts belong to flexible reinforcement [7,8]. Therefore, when cable bolts are used in underground coal mines, they can be inserted into deep rock strata. For cable bolts, the anchorage grout and termination plates can be used to tighten exterior unstable rock strata to deep stable rock strata. Therefore, the self-bearing ability of rock strata around roadways can be fully utilised. Also, the force transfer between the exterior shallow rock strata and the deep stable rock strata can be realised [9].

**Figure 1:**
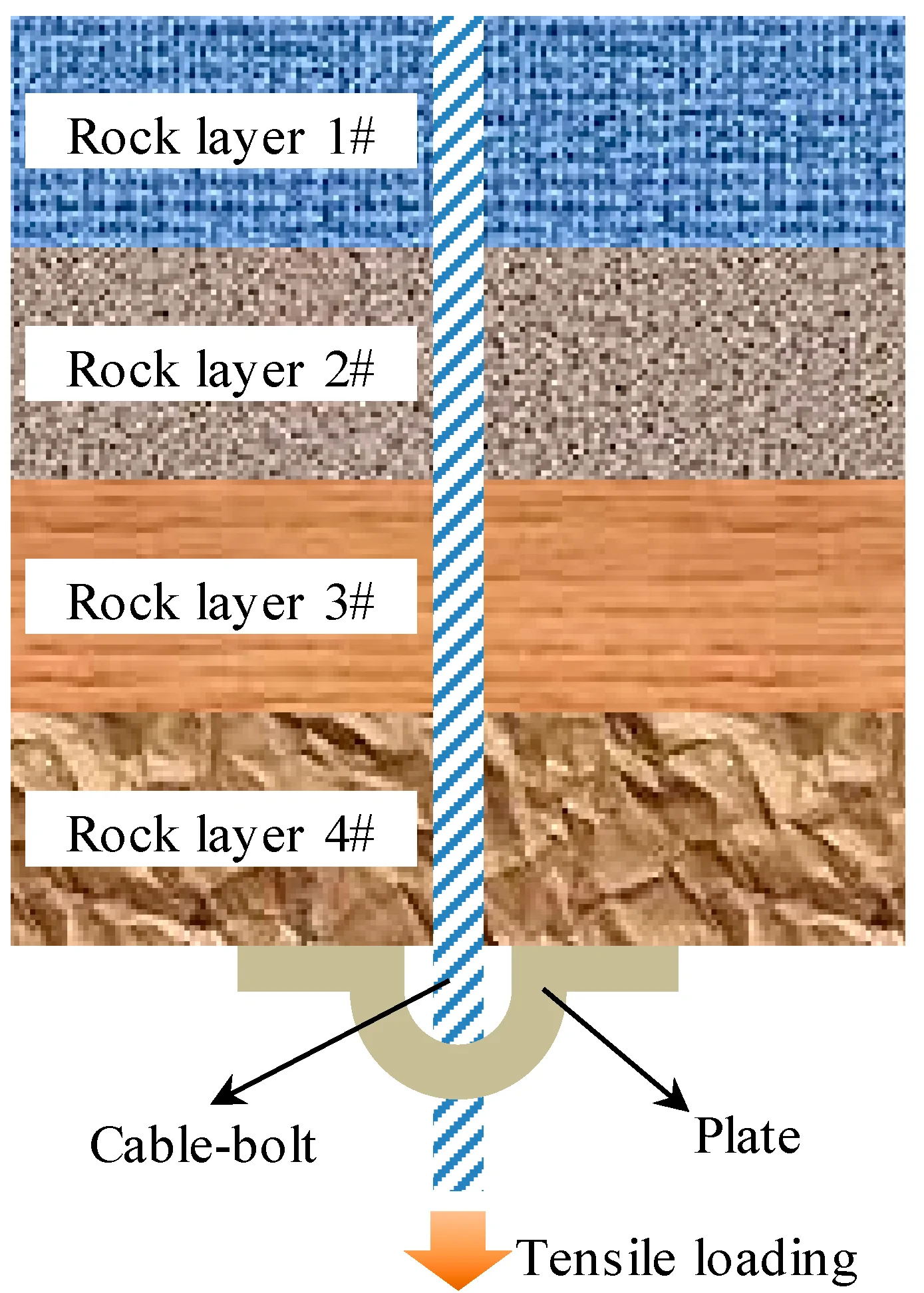
Tensile loading status of the cable-bolt reinforcement in engineering applications.

When rock burst disasters occur, the elastic energy in coal and rock strata will be released suddenly. Then, the elastic energy can be applied on the cable-bolt reinforcement around roadways [10]. Under the impact loading, immediate deformation of coal and rock strata around roadways occurs. Then, the relative deformation between rock strata and cable bolts can occur. This induces shear deformation of the anchorage grout in the cable-bolt reinforcement. Furthermore, this induces loading force on the cable-bolt reinforcement [11]. Under the impact loading, the coal and rock strata around cable bolts can induce tensioning loading (Figure 1), shearing loading, combined tensioning and shearing loading. Consequently, this is likely to induce the anchorage failure of the cable-bolt reinforcement.

To determine the load-bearing behaviour of cable bolts under impact loading, previous researchers conducted research based on a series of methods. These include laboratory tests, theoretical analysis, numerical calculation and in situ tests. This paper aims to review and summarise previous research which focus on the mechanical response and failure mechanisms of the cable-bolt reinforcement under impact loading. When searching for references, the authors used the search engine “scopus”. To search for corresponding references, the authors focused on the research topic of the mechanical response of the cable-bolt reinforcement under impact loading. The academic terms “cable-bolts”, “dynamic response” and “impact loading” were used. The covered period ranged from 1960 to the current. It is noted that when searching for references, it is defined that references that focus on the static performance of cable bolts are excluded.

This paper has two original contributions. First, this paper provides a review on the existing experimental methods that are used to measure the load-bearing behaviour of the cable-bolt reinforcement under the impact loading. Second, this paper provides a review on the mechanical response and failure mechanisms of the cable-bolt reinforcement under the impact loading. Therefore, this paper is beneficial for readers to further understand the mechanical response of the cable-bolt reinforcement under the impact loading.

## 2. Measuring Methods for the Load-Bearing Behaviour of the Cable-Bolt Reinforcement Under the Impact Loading

To study the load-bearing behaviour of the cable-bolt reinforcement under impact loading, previous researchers conducted numerous studies based on laboratory experiments, theoretical analysis, numerical calculation and in situ tests [12]. The advantage of the experimental tests is that the tensile strength and impact strength can be quantitatively evaluated [13].

Among the above research methods, laboratory experiments account for the majority. Compared with theoretical analysis and numerical calculation, when laboratory experiments are used, the load-bearing behaviour of the cable-bolt reinforcement can be directly measured. Additionally, in laboratory experiments, researchers can flexibly change different parameters to simulate different conditions based on research objectives. Then, researchers can investigate the load-bearing behaviour of the cable-bolt reinforcement subjected to impact loading under different conditions. Previous researchers adopted the blasting method in underground discarded roadways in coal mines to simulate the impact loading. The purpose is to compare the difference in the load-bearing behaviour of cable bolts with constant resistance and traditional cable bolts [14]. However, the environment of underground discarded roadways in coal mines is quite complicated. Multiple parameters such as geological conditions are uncontrollable. Therefore, it is not convenient to quantitatively analyse the load-bearing behaviour of the cable-bolt reinforcement under impact loading when different parameters are used.

It is noted that the test standard regarding the load-bearing behaviour of the cable-bolt reinforcement under impact loading is limited. However, it is commonly accepted that to measure the load-bearing behaviour of the cable-bolt reinforcement under impact loading, high velocity should be induced to impact the cable-bolt reinforcement. Following this concept, researchers conducted experimental tests.

According to the loading direction, the laboratory impact-loading tests on the cable-bolt reinforcement can be divided into two categories. They are the horizontal high-speed impact-loading test and vertical drop-hammer impact-loading test. Zhang et al. [15] indicated that previous research mainly focused on the load-bearing behaviour of the cable-bolt reinforcement under static loading. In fact, it cannot exactly reflect the working performance of the cable-bolt reinforcement during the coal-mining process. Based on this issue, they designed a horizontal high-speed impact-loading test. This horizontal high-speed impact-loading test is generally assembled along the horizontal direction. Two hydraulic cylinders were used to apply the rapid loading along reversed directions on the cable bolts. Then, the impact loading was applied on the cable bolts. Its function is used to simulate the impact loading which is induced by dynamic disasters such as roof rupture or rock bursts.

When this horizontal high-speed impact loading method is used, the maximum impact velocity can reach 3 m/s. This velocity is consistent with the maximum roof collapsing velocity in underground coal mines [16]. The maximum impact force can reach 230 kN. The loading duration is usually around three seconds [17]. The boundary condition is that two locking devices are used to fix two horizontal ends of the cable bolt and apply pretension force. However, the limitation is that the impact energy is limited.

When applying the impact loading, the hydraulic cylinders with low flow rate were used. Then, the hydraulic cylinders were used to apply static pretension force on cable bolts. After that, the energy accumulator was used to supply oil for hydraulic cylinders and realise the impact loading. Additionally, during the test process, the high-speed cameras were installed to monitor the impact process of the piston in hydraulic cylinders. Also, the failure modes of the cable-bolt reinforcement can be recorded. Based on the videos and photos recorded by the high-speed cameras, being combined with the data analysis software, the numerical simulation model of the cable-bolt reinforcement under impact loading can be established. Based on this numerical simulation model, the relationship curve between time and impact velocity of the piston can be calculated. The relationship curve between the time and entrance pressure of the hydraulic cylinders can be obtained. Additionally, the relationship curve between the time and impact force of the piston of the hydraulic cylinders can be recorded. Then, the relationship curve between the time and oil volume can be obtained. Based on the above curves, researchers can quantitatively analyse the load-bearing behaviour of the cable-bolt reinforcement under impact loading.

Based on this horizontal high-speed impact-loading test, they conducted preliminary impact-loading tests on cable bolts. The research results indicate that under the impact loading, the hydraulic cylinders can lead to cable-bolt rupture or slippage. Additionally, based on the high-speed camera and simulation analysis, the relationship curve between the time and impact velocity can be obtained. This is beneficial for analysing the load-bearing behaviour of the cable-bolt reinforcement under the impact loading.

This kind of experimental test method has later been used by Yang et al. [18], Zhao et al. [16], Xu et al. [19], Qu et al. [20], Kong and Zhang [21], and Wu et al. [22] to study the mechanical response of the cable-bolt reinforcement.

By contrast, He et al. [23] designed a vertical impact-loading device with a drop hammer. This vertical impact-loading device is basically installed along the vertical direction. This device is primarily composed of the host computer, controlling system and sensor-based system. Based on the component assembly, this vertical impact-loading device is composed of the following parts: the fencing, pulley system, fixed beam, movable beam, hook part, clamping part, hammer, sample, support column, guiding column, protective net, lifting part, buffer and base frame. In this device, the hammer has five different masses: 840 kg, 880 kg, 920 kg, 960 kg and 1000 kg. Therefore, it can meet different impact energy requirements. For the hammer, the maximum dropping height was 1.5 m. An advantage of the vertical drop-hammer impact-loading test is that the impact energy can be calculated conveniently with Equation (1):

(1) E=mgH

where *E*: the impact energy; *m*: the mass of the drop-hammer; *g*: the acceleration of gravity; *H*: the drop height.

However, this impact energy calculation method also has limitations. First, during the drop test process, the air friction will induce impact energy loss and this influence is not considered. Second, when impact events occur, the hammer will generate elastic deformation and absorb certain impact energy. This absorbed impact energy is also not considered.

According to the calculation method based on the hammer weight, for this vertical impact-loading device, the impact energy is around 12,600 J–15,000 J.

When the vertical drop-hammer impact-loading method is used, the impact velocity can reach 11 m/s [24]. As for the impact energy, it can reach 60,000 J [25]. The boundary condition is that the bottom end is fixed to the ground and the top hammer is free to drop. The limitation is that during the impact process, the static weight is also applied on the cable-bolt reinforcement.

During the test process, the top terminal of cable bolts can be fixed to the top of this vertical impact-loading device. After that, the hammer is lifted to a certain position that is away from the tested plate. The lifting velocity can be increased from zero to 3 m/min. After that, the hammer is released. Then, it can impact the plate. Following this, the hammer can be lifted again to the certain altitude. Then, the cyclic impact loading can be conducted. This cyclic impact loading can be conducted until cable-bolt rupture or cable-bolt slippage.

For this vertical impact-loading device, the measuring and analysis software of DASP is equipped. It can record the real-time data during the hammer-dropping process. Additionally, this vertical impact-loading device is equipped with pressure transducers. This pressure transducer has a circular geometry. In this pressure transducer, there are two groups of quartz crystal sheets, electrical sheets, force plates, shells and sockets. For this pressure transducer, the minimum detection precision is 0.025 N. It can accurately measure the variation process of the force after cable bolts are subjected to impact loading. Based on the above data-monitoring system, researchers can obtain the single impact resistance and single deformation of cable bolts subjected to impact loading. Then, after multiple instances of impact loading, the relationship curves between the impact resistance, deformation of cable bolts and impact quantity can be obtained. Then, under different impact energies, the variation rule of the cable-bolt load-bearing behaviour can be analysed.

Based on this method, He et al. [26] took cable bolts with constant resistance as the research objectives. The purpose is to confirm the reliability of this vertical impact-loading device. The research outcomes indicate that the designed vertical impact-loading device can realise the cyclic impact loading on cable bolts. Therefore, this device can be used to study the anti-impact performance of cable bolts under different impact energies.

**Figure 2:**
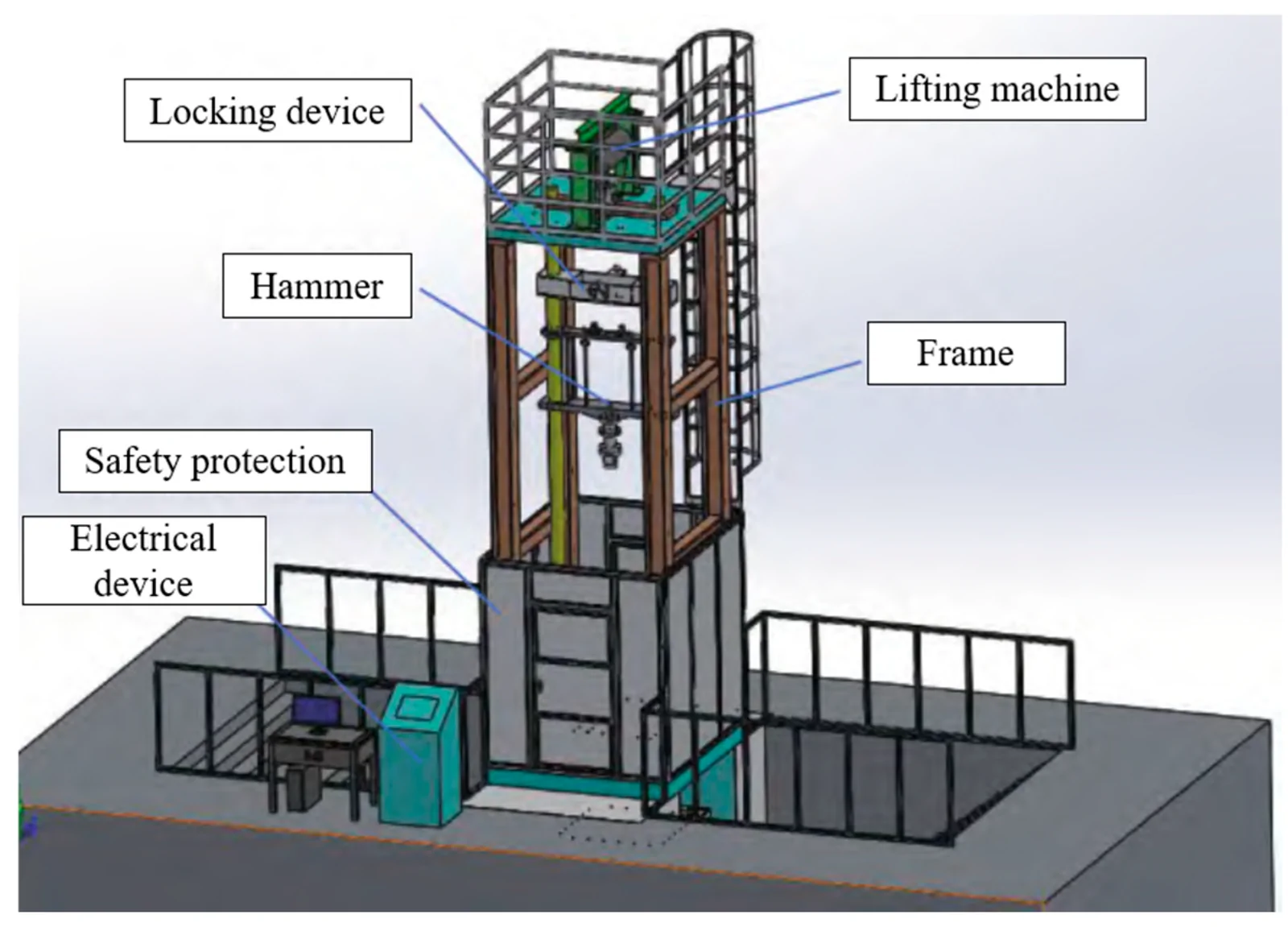
The vertical drop-hammer impact loading device [2].

This vertical impact-loading method has been used by different researchers. For example, Wu et al. [2] developed a vertical impact-loading device with a maximum impact energy of 30,000 J (Figure 2). This device can be used to measure the dynamic response of various kinds of materials, including cable bolts, plates and steel meshes. Additionally, this vertical impact-loading device can be used to impact the similarity model of the anchorage body. This vertical impact-loading device can apply an impact velocity of 3 m/s–8 m/s on anchorage elements, such as cable bolts. Therefore, this device can effectively simulate the impact velocity of the rock masses around roadways when rock burst disasters occur.

Additionally, in the university of Wollongong, Khaleghparast et al. [27] developed a vertical drop-hammer impact-loading device. The hammer height is 4 m and its mass is 592 kg. The maximum impact velocity is 9 m/s. It can effectively simulate the eject velocity of rock masses when rock bursts occur. In this device, a dynamic pressure transducer with a measuring range of 1200 kN is embedded. Its purpose is to measure the impact force when the hammer contacts the impactor. If the frictional loss during the hammer impact process is neglected, the maximum impact energy is 23,000 J. This device is also equipped with the high-speed camera Memrecam HX-7. It can record the high-speed impact process with a maximum recording rate of 0.2 million frames per second. Wu et al. [28] designed a vertical impact-loading device. For their device, the maximum impact height is 14 m. The hammer mass can be changed between 30 kg and 1030 kg. Wang et al. [25] designed a static and dynamic coupled test system for anchorage samples. This test system is composed of a hammer whose mass is 3000 kg. The hammer can be lifted to 2 m high. Then, it can be released and it can impact the anchorage sample of rock (cable) bolts. This test system can generate around 60,000 J energies. Therefore, it can effectively simulate impact energies with different grades.

The comparison between the horizontal high-speed impact-loading method and vertical drop-hammer impact-loading method is summarised in Table 1.

## 3. Mechanical Response of the Cable-Bolt Reinforcement Under Impact Loading

When rock bursts occur, intensified impact waves can occur. Then, the impact waves can rapidly propagate towards rock masses around roadways. When the cable-bolt reinforcement is applied on rock masses around roadways, cable bolts can be installed in coal and rock masses around roadways along different angles. Therefore, induced by rock bursts, the impact waves can be applied on the cable-bolt reinforcement along different directions, such as axial direction, transverse direction and intersected direction. Consequently, the impact waves can induce tensioning effect, shearing effect, and coupled tensioning and shearing effect on the cable-bolt reinforcement [29].

Cable bolts belong to flexible reinforcement elements. Under static loading, the shear strength of cable bolts is generally smaller than the tensile strength. Specifically, the shear strength of cable bolts is generally 0.6–0.8 times of the tensile strength [30]. Currently, the impact tests on the cable-bolt reinforcement mainly focus on the tensile response of cable bolts. Much less work has been conducted to study the shear response of cable bolts.

It is noted that in practical engineering cases, after rock bursts occur, the cable-bolt reinforcement is more likely to encounter the coupled tensioning and shearing impact loading. This is because in practical engineering cases, rock masses are composed of multiple rock joints. After rock bursts occur, the cable-bolt reinforcement may deform along the axial direction of cable bolts and shear direction of rock joints. However, in laboratory tests, quite little research has been conducted to evaluate the mechanical response of the cable-bolt reinforcement under the coupled tensioning and shearing loading condition. The experimental challenge is that during the short loading period in the impact-loading test, the tensioning force and shearing force should be applied on the cable-bolt reinforcement simultaneously. This makes it quite complicated to realise this experimental test.

**Figure 3:**
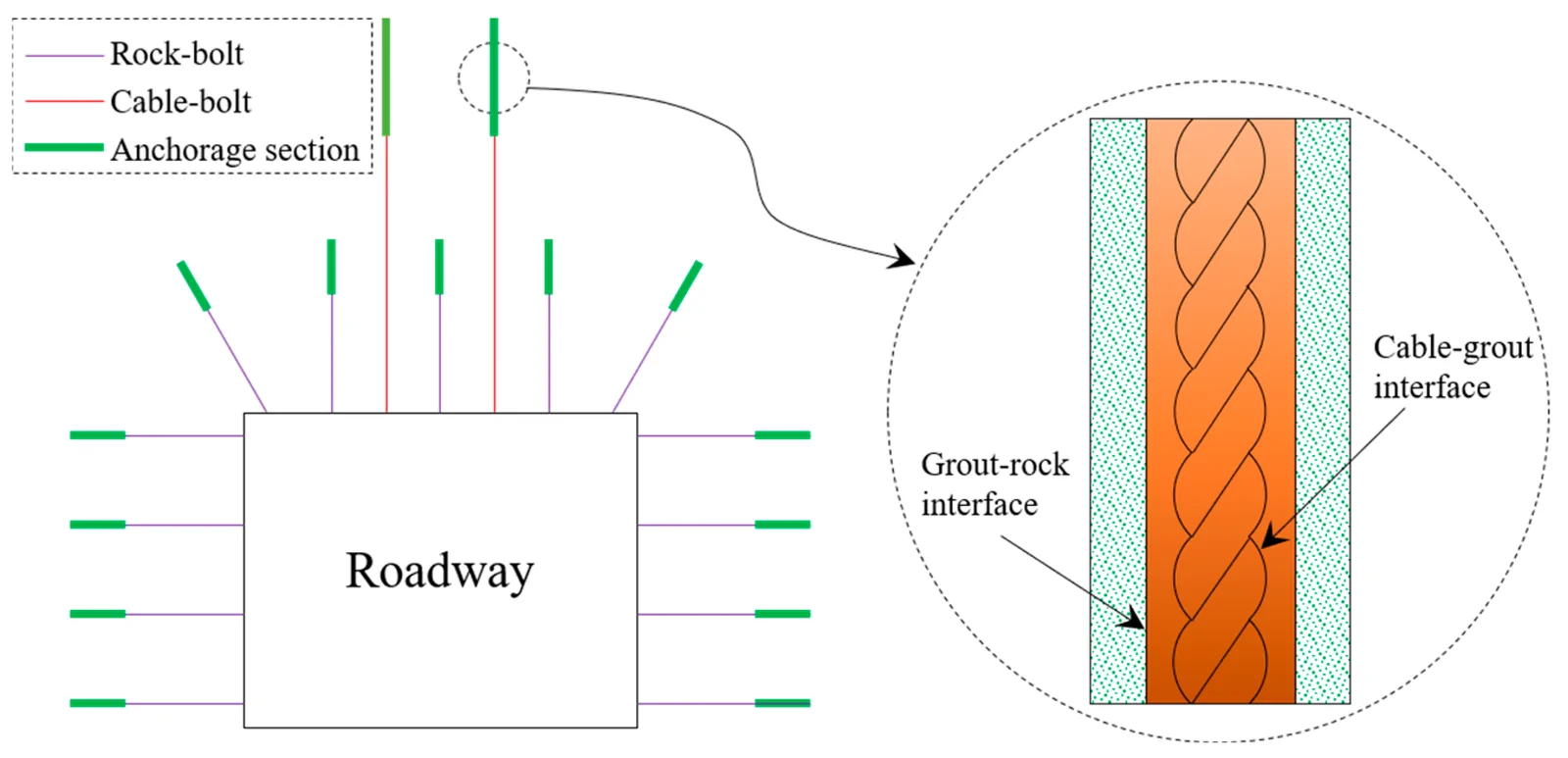
The interaction between cable bolts, anchorage grout and surrounding rock masses.

When cable bolts were used, they were usually combined with the anchorage grout and surrounding rock masses to form the cable-bolt reinforcement [31]. The mechanical response of the cable-bolt reinforcement is totally different from that of pure cable bolts. This is because the mechanical response of the cable-bolt reinforcement was directly influenced by multiple parameters, such as the cable bolts, interfaces [32], anchorage grout and rock masses (Figure 3). When the impact loading was applied, their mechanical response was different since their materials were different. After the impact loading occurred, the longitudinal deformation of cable bolts, anchorage grout and surrounding rock masses was different since their elastic deformation was different. Consequently, slippage occurred between the cable bolts and surrounding rock masses. This led to shear deformation of the anchorage grout in boreholes. Then, the bond stress is generated in the anchorage grout. Consequently, the force transfer can be realised between cable bolts and surrounding rock masses via the anchorage grout.

Under the in situ condition, the impact loading may be applied on the cable-bolt reinforcement along the tensioning direction and shearing direction. This will induce the tensile and shear load-bearing behaviour of the cable-bolt reinforcement. Therefore, in the following section, the load-bearing behaviour of the cable-bolt reinforcement is summarised in terms of the tensioning direction and shearing direction.

In the following section, when evaluating the mechanical response of the cable-bolt reinforcement, this paper mainly uses three parameters, including impact energy, impact force and impact deformation. The impact energy represents the energy that is applied by the cable-bolt reinforcement. The impact energy can directly determine the failure status of the cable-bolt reinforcement. The impact force indicates the force that is applied on the materials in the cable-bolt reinforcement. The impact force will induce stress in the materials in the cable-bolt reinforcement. Once the induced stress is beyond the strength of the materials in the cable-bolt reinforcement, it will also lead to failure of the materials in the cable-bolt reinforcement. The impact deformation indicates the deformation of materials in the cable-bolt reinforcement. Under the impact loading, when the materials in the cable-bolt reinforcement deform, they will absorb the impact energy. Therefore, there is a close relationship between impact energy, impact force and impact deformation. Then, this paper used these three parameters to evaluate and judge the mechanical response of the cable-bolt reinforcement under the impact loading.

### 3.1. Tensile Load-Bearing Behaviour of the Cable-Bolt Reinforcement

(1) Cable-bolt structure

Wu et al. [2] conducted impact-loading tests on traditional cable bolts whose diameter is 21.8 mm and tensile strength is 1860 MPa. Results showed that under static loading, for this cable bolt, the rupture force was 538 kN. After cable-bolt rupture, the elongation rate was 5%. However, under the impact loading, for this cable bolt, the rupture force was relatively larger than 538 kN. After cable bolts ruptured, the elongation rate dramatically reduced to 2.5%. This indicates that under impact loading, the elongation of traditional cable bolts is relatively poor. Consequently, traditional cable bolts are likely to break. This is consistent with the in situ cable-bolt rupture which is induced by the impact loading during the underground coal-mining process.

It is generally accepted that the anti-impact response of traditional cable bolts is relatively poor and the cable-bolt structure directly influences the tensile load-bearing behaviour of cable bolts. Based on this concept, researchers optimised the cable-bolt structure. Then, multiple cable bolts that have anti-impact performance are designed. In the laboratory, Anzanpour et al. [33] cast the traditional cable-bolt anchorage body and bulbed cable-bolt anchorage body. Based on impact-loading tests, they compared the mechanical performance difference between the traditional cable-bolt anchorage body and bulbed cable-bolt anchorage body. Their results showed that under the impact loading, compared with traditional cable bolts, the bulbed cable-bolt anchorage body can show another 40% of the bearing capacity. This indicates that the bulbed structure can apparently improve the bearing capacity of cable bolts under the impact loading. Additionally, compared with the static loading, when the impact loading occurred, the energy that is needed to pull cable bolts out from the cable-bolt anchorage body will reduce by 20–50%.

**Figure 4:**
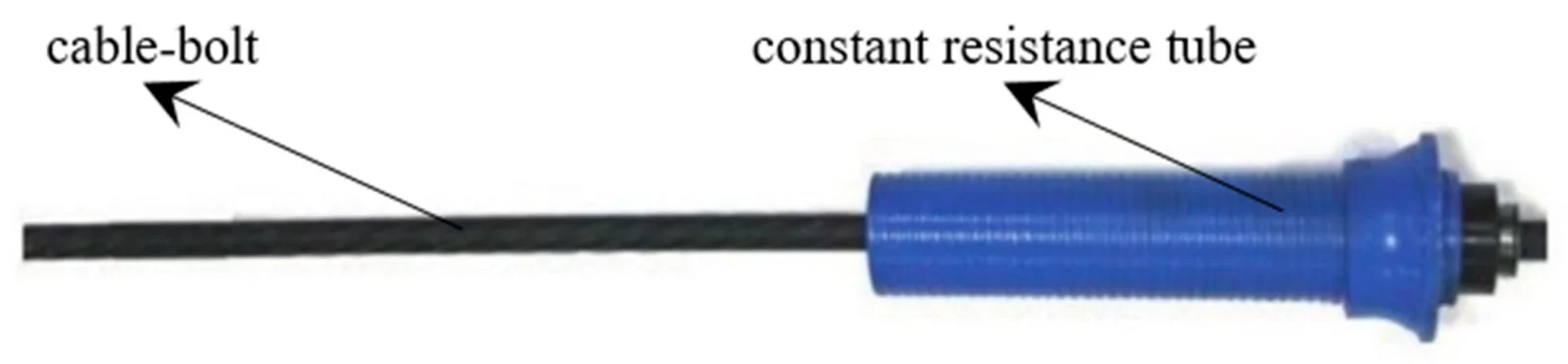
Diagram of the cable bolt with constant resistance [26].

He et al. [26] designed the cable bolt with constant resistance, which is composed of the cable-bolt strand, constant-resistance tube, and plate and locking device, as shown in Figure 4. This cable bolt with constant resistance had a diameter of 21.8 mm. They conducted repeated impact-loading tests on this cable bolt and the number of repeated times was ten. They found that when the number of impact times increased from one to two, the single pulling displacement decreased from around 60 mm to around 45 mm. After that, when the number of impact times increased from two to ten, the single pulling displacement generally increased from around 45 mm to around 53 mm. The impact resistance of this cable bolt was also monitored. When the number of impact times increased from one to three, the impact resistance of cable bolts decreased from around 390 kN to around 330 kN. After that, the oscillation of the impact resistance occurred. When the number of impact times increased from five to ten, the impact resistance of cable bolts generally increased.

The credibility of this cable bolt with constant resistance was also confirmed with in situ tests. For example, He et al. [23] conducted in situ impact-loading tests on cable bolts with constant resistance. The blasting method was used to simulate rock burst events. Their results showed that if roadways were reinforced with traditional cable bolts, roof collapsing failure occurred. However, if roadways were reinforced with cable bolts with constant resistance, limited deformation of roadways occurred. After two blasting events, the cable bolts with constant resistance showed consistent deformation ability, which was consistent with the results obtained with laboratory tests.

**Figure 5:**
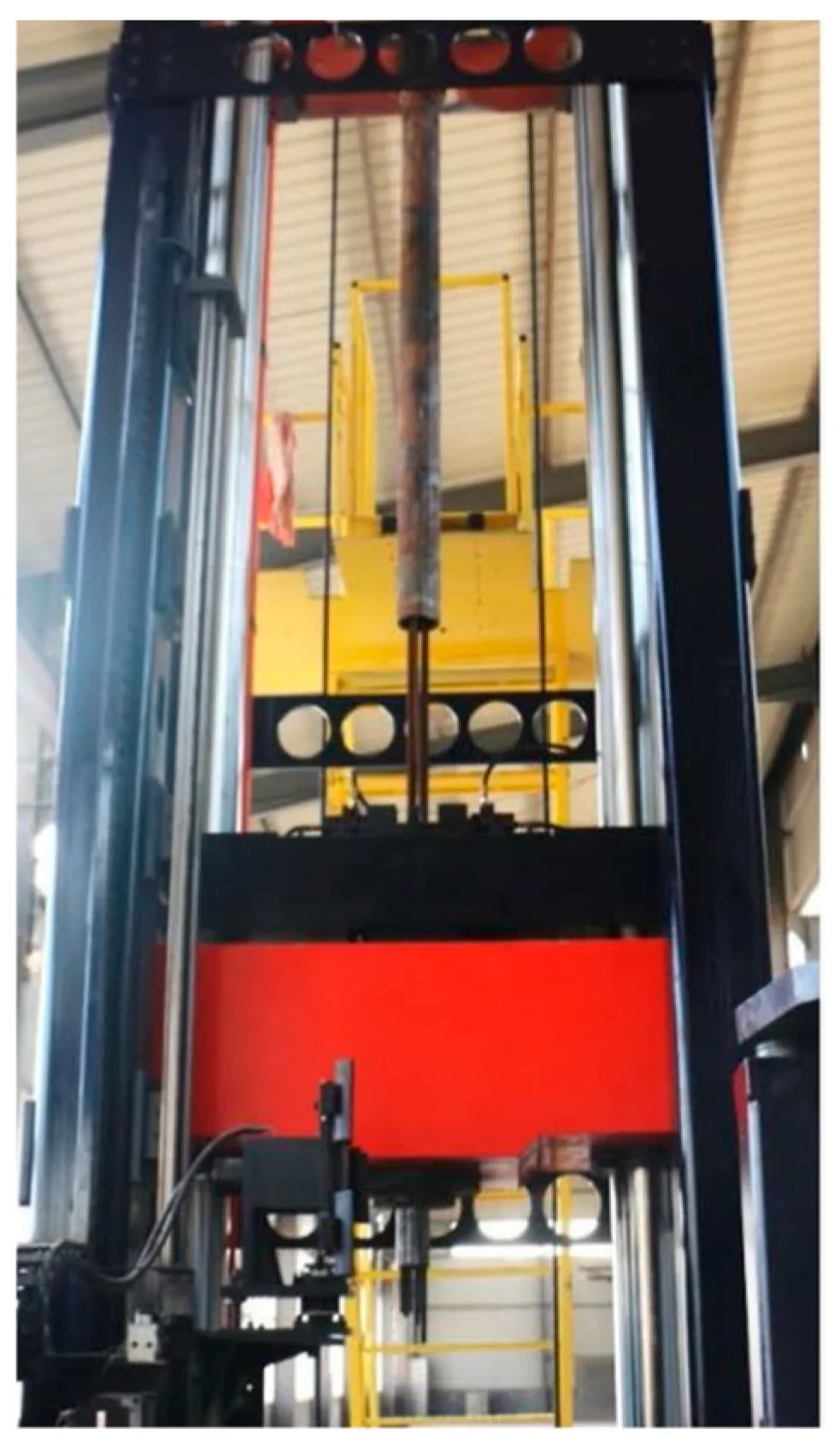
Laboratory impact-loading tests on cable bolts with constant resistance [34].

The above analysis showed that laboratory test results can provide theoretical guidance on in situ application of cable bolts with constant resistance. In fact, the laboratory tests and in situ tests proved that for each instance of impact loading, cable bolts with constant resistance can uniformly absorb impact energy. Therefore, this cable bolt shows relatively better anti-impact performance. More importantly, the anti-impact performance of this cable bolt was later validated by multiple experimental tests [34,35], as shown in Figure 5.

He et al. [14] analysed the geometric functioning relationship between the constant-resistance tube and cable bolts after the impact loading. Based on the rheological mechanical model, they established an elastic perfectly plastic constitutive model which can reflect the working performance of cable bolts with constant resistance. Therefore, from the perspective of theoretical analysis, they analysed the relationship between force and displacement after this cable bolt was loaded.

Zhang et al. [36] indicated that after the cable bolt with constant resistance was subjected to impact loading, the ultimate elongation can reach 1.6 m. The bearing capacity of this cable bolt was around 280 kN~375 kN. When the loading force was beyond the constant resistance force, the cable bolt entered the slippage period. Additionally, the temperature in the constant-resistance tube increased dramatically. After that, the temperature decreased gradually. Then, the temperature in the constant-resistance tube became stable. During the whole loading process, the maximum temperature in the constant-resistance tube was 35.6 Celsius degrees. They also measured the lateral deformation and axial deformation of the cable-bolt reinforcement. Through dividing the lateral deformation by the axial deformation, they obtained the Poisson’s ratio. They found that the Poisson’s ratio was −0.5~−2.0. Therefore, the constant-resistance tube showed apparent negative Poisson’s effect. For the external diameter of the constant-resistance tube, the maximum increment was 4.1 mm.

Hu et al. [37] developed the true tri-axial rock burst test rig. Relying on it, they conducted tests on rock samples which were reinforced with the similarity simulation model of the cable bolt with constant resistance. Compared with the no-support condition, when cable bolts with constant resistance were used, after the rock broke, the energy-releasing speed became slow. This dramatically reduced the possibility of rock bursts. From the perspective of theoretical analysis, this study explained the possibility that the cable bolt with constant resistance can control the surrounding rock-mass failure extent when rock burst occurs.

Wang et al. [38] utilised the energy-absorbing ability of the hexagonal tube which is based on squeezing and friction. They designed an anti-impact rock (cable) bolt which is composed of the energy-absorbing tube, cable bolts and energy-absorbing device. They analysed the geometric dimension of the energy-absorbing tube and deformation performance of the energy-absorbing rock (cable) bolts. From the perspective of mechanical analysis, the squeezing and deformation process of the hexagonal tube was clarified. After the frictional column fully enters the hexagonal tube, the energy-absorbing rock (cable) bolt can show the constant resistance ability. Additionally, they conducted impact-loading tests on energy-absorbing and anti-impact rock (cable) bolts [3]. Their results showed that the energy-absorbing and anti-impact rock (cable) bolts showed relatively better adaptive impact response. When the impact energy was relatively small, the bearing performance curves of the energy-absorbing and anti-impact rock (cable) bolt only showed the increasing period of the initial longitudinal force and the stable longitudinal force period. The energy-absorbing tube showed stable energy-absorbing performance. When the impact energy is relatively large, the load-bearing curves of the energy-absorbing and anti-impact rock (cable) bolt showed the rapid increase of the longitudinal force and then its stable period. The energy-absorbing tube first absorbed part of the energies. After that, the bolt absorbed the residual energy through the plastic elongation. Additionally, for these anti-impact rock (cable) bolts, the advantages are that the material and structural parameters of the energy-absorbing device can be adjusted according to practical cases. This is beneficial for adapting to different types of cable bolts. Also, this can change the energy-absorbing resistance of the anti-impact cable bolts from 300 kN to 500 kN. Therefore, it can meet the reinforcement requirements under different working situations [39].

Fu [40] optimised the manufacturing technique of cable bolts. Special heat treatment was conducted on traditional steels. The carbon diffusion was added between the quenching process and tempering process. The function was to improve the toughness and elongation rate of cable bolts. Based on this technique, they designed the cable bolt with high impact toughness. This cable bolt had a structure of 1 × 19 wires and its diameter was 21.8 mm. The lay pitch of this cable bolt was 225 mm and the strength was beyond 1770 MPa. The maximum elongation rate was 8%. Through comparing the impact force–displacement curves of the traditional cable bolts and cable bolts with high impact toughness, they found that for these two types of cable bolts, the rupture force was around 524 kN. Therefore, there was a marginal difference between them. However, when entering the stable deformation period, the maximum deformation of traditional cable bolts was zero. Compared with that, for cable bolts with high impact toughness, the maximum deformation was 42.1 mm. This indicates that under impact loading, after traditional cable bolts entered the stable deformation period, they did not have the continuous deformation ability. By contrast, when cable bolts with high impact toughness entered the stable deformation period, they can absorb impact energy based on continuous deformation. Therefore, the designed cable bolts with high impact toughness had strong anti-impact ability. When rupture occurred, the absorbed impact energy was 2.67 times of traditional cable bolts.

Based on the squeezing and deformation principle of steel tubes, Wu et al. [28] designed a cable bolt with high constant resistance which belonged to the concentric tube type. This cable bolt is composed of the following parts: the steel cable strand, restraint ring, compression tube, plate, nut and locking device. Before the deformation of the surrounding rock masses, the compression tube was in the original length state. After deformation of surrounding rock masses around roadways, the compression tube was subjected to squeezing and compression. Then, it entered the restraint ring. Based on the frictional function between the compression tube and restraint ring, the reinforcement resistance was supplied and impact energy was consumed. When the surrounding rock mass deformation was beyond the length of the compression tube, the compression tube stopped deformation. Additionally, the internal force of cable bolts increased dramatically. When it reached the tensile strength of cable bolts, the cable-bolt rupture occurred.

After impact-loading tests were conducted on this cable bolt, the following results were obtained. When impact events occurred, after 3 ms, the compression tube was subjected to impact loading and plastic deformation occurred. Then, the impact kinetic energy can be consumed. When impact events occurred, after 42 ms, the compression tube reached the maximum deformation. Repetition tests showed that after impact events occurred, this cable bolt can reduce the impact force dramatically to zero within 50 ms. The buffering ability was stable and this cable bolt had relatively better energy-absorbing effect.

Jia et al. [41] designed a composite cable bolt with a constant-resistance tube. This constant-resistance tube can be coupled with traditional cable bolts. Its components include the coned locking nut, plate and termination plate. The resistance provided by this constant-resistance tube includes the following two periods: the rapid resistance ascending period and stable resistance period. In practical applications, the stable resistance period accounts for 85%~90% of the whole test process. Therefore, this constant-resistance tube can provide stable working resistance. It can realise providing stable reinforcement resistance during the large deformation process of surrounding rock masses around roadways.

(2) Impact energy

Cheng et al. [42] studied the influence of impact energy on the dynamic mechanical response of cable bolts. They took the traditional cable bolts which are commonly used in typical rock burst mines as the research objectives. Then, they conducted impact-loading tests. Their results showed that with increasing impact energy, the maximum impact force of cable bolts increased gradually. When the impact energy was larger than 15,000 J and smaller than the peak energy, cable-bolt rupture may occur. With the impact energy increasing, the plastic deformation of cable bolts increased rapidly. The impact energy gradually changed to the deformation energy of cable bolts. Additionally, during the impact process, there was a positive relationship between the energy absorbed by cable bolts and the plastic deformation of cable bolts.

(3) Pretension force

Wang et al. [25] compared the anti-impact performance of pretensioned cable bolts and untensioned cable bolts. In their tests, the cable bolt is composed of six steel wires. Its tensile strength was 1860 MPa. The cable-bolt diameter is around 22 mm and the whole length is 3.5 m.

The vertical drop-hammer impact-loading test was used. The weight of this hammer was 2000 kg and the drop height was 500 mm. During testing, a maximum impact energy of 10,000 J was applied on the cable-bolt reinforcement. During the impact process, they set six different pretension forces, including 0, 150 kN, 180 kN, 210 kN, 250 kN and 300 kN. Compared with untensioned cable bolts, after the pretension force was applied, the anti-impact resistance of cable bolts significantly reduced. When the pretension force ranged between 150 kN and 300 kN, compared with the untensioned condition, the impact energy that the high-strength cable bolts can resist will be reduced by 76.5%~84.6%.

(4) The coupling relationship between the surface protecting and cable bolts

In engineering practices, cable bolts are usually coupled with plates, a W-shaped steel panel and metal diamond-shaped mesh. He et al. [43] studied the mechanical response of plates and a W-shaped steel belt under the impact loading. They took the plate whose material was Q235 and edge length was 300 mm as the research objective. They also took the W-shaped steel panel whose material was Q235 and dimension was 450 mm × 280 mm as the research objective. Under the impact loading, the plate and the W-shaped steel panel showed the three-period load-bearing behaviour. These three periods include the dramatic loading, vibrated stable loading and rapid unloading. With the impact energy increasing, the maximum impact force and vibrated magnitude of the plate increased significantly. Under the single impact loading, the impact period of the plate was less than 8 ms.

Wu et al. [2] conducted impact tests on the steel diamond-shaped mesh. This steel diamond-shaped mesh was fabricated with 8# iron wires whose diameter was 4.06 mm. The dimension of the mesh hole was 50 mm × 50 mm. The dimension of the diamond-shaped mesh was 1.2 m × 1.2 m. The results showed that with the increasing of the impact energy, the maximum impact force of the steel diamond-shaped increased gradually. For the steel diamond-shaped mesh, the deformation increased first and then reduced. Under the impact loading, the steel diamond-shaped mesh had relatively better buffering performance.

(5) Spark effect

After cable bolts are subjected to impact tension, during the slippage and rupture process, frictional sparks may occur along the free length of the cable bolts. When coal mines exploit coal resources, gas emission will occur from coal and rock masses. Therefore, after cable bolts are subjected to impact loading, whether the induced frictional sparks will lead to gas explosion should be determined. Then, Qu et al. [20] studied the generation mechanisms of frictional sparks which are induced by the free length of the cable bolts after impact loading. Their study showed that if the quality of cable bolts met the requirement, during the loading process, it was not likely to induce frictional sparks. However, considering the complexity of engineering cases, due to the fact that the loading force between steel wires in cables is not uniform or the clamping plates are not even, slippage of partial steel wires may occur. This may even induce frictional sparks.

Wu et al. [22] conducted impact-loading tests on three types of cable bolts—the cable bolts with diameters ranging from 15.24 mm to 21.6 mm. During the test process, it is confirmed that the gas concentration and the oxygen concentration met the gas explosion condition. Then, the infrared thermal imager was used to observe the frictional spark temperature during the test process. Their results showed that no matter how the cable bolts failed, the maximum temperature of frictional sparks induced by loading of cable bolts was obviously smaller than the minimum temperature that was needed for the gas explosion. Additionally, the existing period of frictional sparks was much smaller than the induction period of the gas explosion. Therefore, they concluded that the frictional sparks induced by the cable-bolt rupture will not induce the gas explosion. Nevertheless, this conclusion is limited to the currently obtained laboratory tests. Whether the results are applicable to in situ cases still needs to be studied.

### 3.2. Shear Load-Bearing Behaviour of the Cable-Bolt Reinforcement

It has been noted that in engineering practices, the impact wave induced by rock bursts may be applied on the cable-bolt reinforcement along the transverse direction. Then, the impact wave may induce shear function on the cable-bolt reinforcement. Therefore, researchers conducted a study to analyse the shear load-bearing behaviour of the cable-bolt reinforcement under the impact loading.

Ortlepp and Erasmus [44] designed a wave-shaped cable bolt with a high elongation rate. Then, they conducted impact shear tests on cable bolts. During testing, they used a wedge-shaped hammer to impact cable bolts with a velocity of 11 m/s. Their results showed that when the cable-bolt elongation was less than 220 mm, there was no apparent decreasing of the bearing capacity. During testing, the cable bolt can provide a maximum value of 200 kN to resist the shear force.

Considering that the experimental shear tests on the cable-bolt reinforcement is relatively complicated to perform, Tahmasebinia et al. [45] indicated that numerical simulation can be an efficient approach. Following this concept, they used the finite-element method to create a numerical model of the cable-bolt reinforcement. The commercial software Abaqus was used. It is assumed that the interface behaviour between rock masses and cable bolts can be properly simulated with the surface-to-surface interaction in Abaqus. Based on this finite-element simulation, they found that after the impact loading occurred, the momentum energy was initially transferred to the rock-mass surface. Then, the momentum energy was transferred to the cable bolts. Additionally, they found that the cable-bolt reinforcement failure started with a deformed shape. After that, brittle shear failure occurred. The credibility of the simulation results was confirmed with experimental tests.

Following this approach, they used the numerical simulation to study the mechanical response of the cable-bolt reinforcement under different impact loading [46]. In this case, they created more than five hundred simulation cases. They found that with the increasing of the cable-bolt diameter and cable-bolt yield strength, the resistance that the cable-bolt reinforcement can provide increased apparently.

Wu et al. [47] compared the bearing capacity of three types of cable bolts under the impact loading. During testing, the traditional cable bolts, energy-absorbing cable bolts with a capacity of 160 kN and energy-absorbing cable bolts with a capacity of 350 kN were used. Their results showed that compared with traditional cable bolts, the energy-absorbing cable bolts can resist the impact-loading force for a long period. After the impact loading, the vibration range was relatively small. Under multiple instances of impact loading, for the energy-absorbing cable bolts, the constant-resistance tube can deform stably. Therefore, it can provide stable working resistance. Therefore, this type of cable bolt had superior anti-shearing capacity.

The above analysis showed that previous researchers commonly accepted that traditional cable bolts did not have enough anti-impact ability. Under impact loading, the bearing capacity of traditional cable bolts was poor. Once the impact loading occurred, traditional cable bolts cannot show their full elongation ability. Experimental results showed that once the impact loading occurred, the elongation rate of traditional cable bolts reduced to only half of the elongation rate of traditional cable bolts under static loading [2].

To solve this issue, previous researchers proposed different approaches. Some researchers found that modifying the geometry of the traditional cable bolts can improve the anti-impact ability of cable bolts. For example, adding bulbed geometry can increase the maximum bearing capacity of cable bolts [33]. Other researchers found that creating a composite structure composed of cable bolts and constant-resistance tubes can significantly improve the anti-impact ability of cable bolts. Typical examples included the cable bolt with constant resistance [26] and the anti-impact rock (cable) bolt [38]. Additionally, certain researchers found that optimising the manufacturing technique of cable bolts can improve the anti-impact performance of cable bolts. For example, the designed cable bolts with high impact toughness showed superior anti-impact ability compared with traditional cable bolts [40].

Additionally, the mechanical response of the cable-bolt reinforcement is influenced by multiple parameters. For example, larger impact energy may lead to rupture of the cable bolts directly. Then, the pretension force can also influence the mechanical response of the cable bolts under impact loading. However, the results showed that with the increasing of the pretension force, the anti-impact resistance of cable bolts reduces. When cable bolts are used in engineering cases, they are usually coupled with surface-protecting instruments, such as plates, steel panels and meshes. Experimental results showed that the interaction between cable bolts and surface-protecting instruments can affect the mechanical response of the cable-bolt reinforcement. Researchers also paid attention to the sparking effect of the cable bolts subjected to the impact loading. It is originally inferred that during the impact-loading process of the cable bolts, sparks may generate because of the sudden tensile rupture of the cable bolts. Whether the induced sparks will lead to a gas explosion in underground coal mines is concerning. However, the experimental test results showed that the temperature of the induced sparks is much smaller than the temperature that is needed to trigger a gas explosion.

These studies provided new approaches and instruments to prevent failure of the cable-bolt reinforcement under impact loading. However, most of the current research is focused on the mechanical response of the cable bolts under impact loading. In reality, the cable bolts are coupled with surrounding rock masses via the anchorage grout. The limitation is that little research has been paid to the mechanical response of the system composed of the cable bolts, anchorage grout and surrounding rock masses under impact loading.

## 4. Failure Modes and Failure Mechanisms of the Cable-Bolt Reinforcement

When the impact force is applied on cable bolts and plates, there are basically three failure modes: cable-bolt rupture, cable-bolt slippage and locking-nut slippage [19]. Under impact loading, if the dynamic tensile strength of cable bolts is not sufficient enough, the cable-bolt rupture may occur. This is more likely to occur when the anchorage length of cable bolts is beyond the critical anchorage length. The critical anchorage length is defined as the anchorage length of cable bolts when the cable bolts reach their tensile limit [17]. The critical anchorage length of cable bolts is closely related to the test environment. In fact, the critical anchorage length of cable bolts can be influenced by multiple parameters, including the cable-bolt type, grout type, grout strength, rock-mass strength and surrounding stress levels.

If the anchorage length is too long, the frictional resistance induced by the mechanical interlock and friction is easily larger than the tensile strength of cable bolts. Under impact loading, after large deformation of rock masses occurs, the cable-bolt rupture may occur in the free length of cable bolts. This indicates tensile failure of the cable bolts.

Additionally, if the bond between the cable bolts and anchorage grout is weak, the cable bolt may be pulled out from the anchorage grout. This indicates cable-bolt slippage failure. This is mainly induced by the insufficient bond strength of the cable–grout interface. It should be noted that previous research indicates that when the rock masses around roadways belong to weak rock masses, slippage failure may occur at the grout–rock interface.

Then, after the locking nuts are installed on the terminal of cable bolts, if the frictional resistance between the cable bolts and locking nuts is weak, slippage between the cable bolts and locking nuts may occur. This indicates locking-nut slippage failure.

Additionally, the failure modes of the cable-bolt reinforcement are directly related to the pretension force [20]. Specifically, Qu et al. [20] conducted impact-loading tests on traditional cable bolts. In their testing, the horizontal high-speed impact-loading device was used to apply the impact loading on cable bolts. The maximum impact force was 230 kN and the impact period was three seconds. During testing, three different pretension levels were applied: 30 kN, 50 kN and 90 kN. Test results showed that when the pretension level was less than 90 kN, either slippage or rupture of cable bolts occurred. However, when the pretension level was 90 kN, only rupture of cable bolts occurred. It is noted that the results were not generalised. This means that the failure mode was closely related to the test environment. Once the test environment is changed, the corresponding failure mode may change.

**Figure 6:**
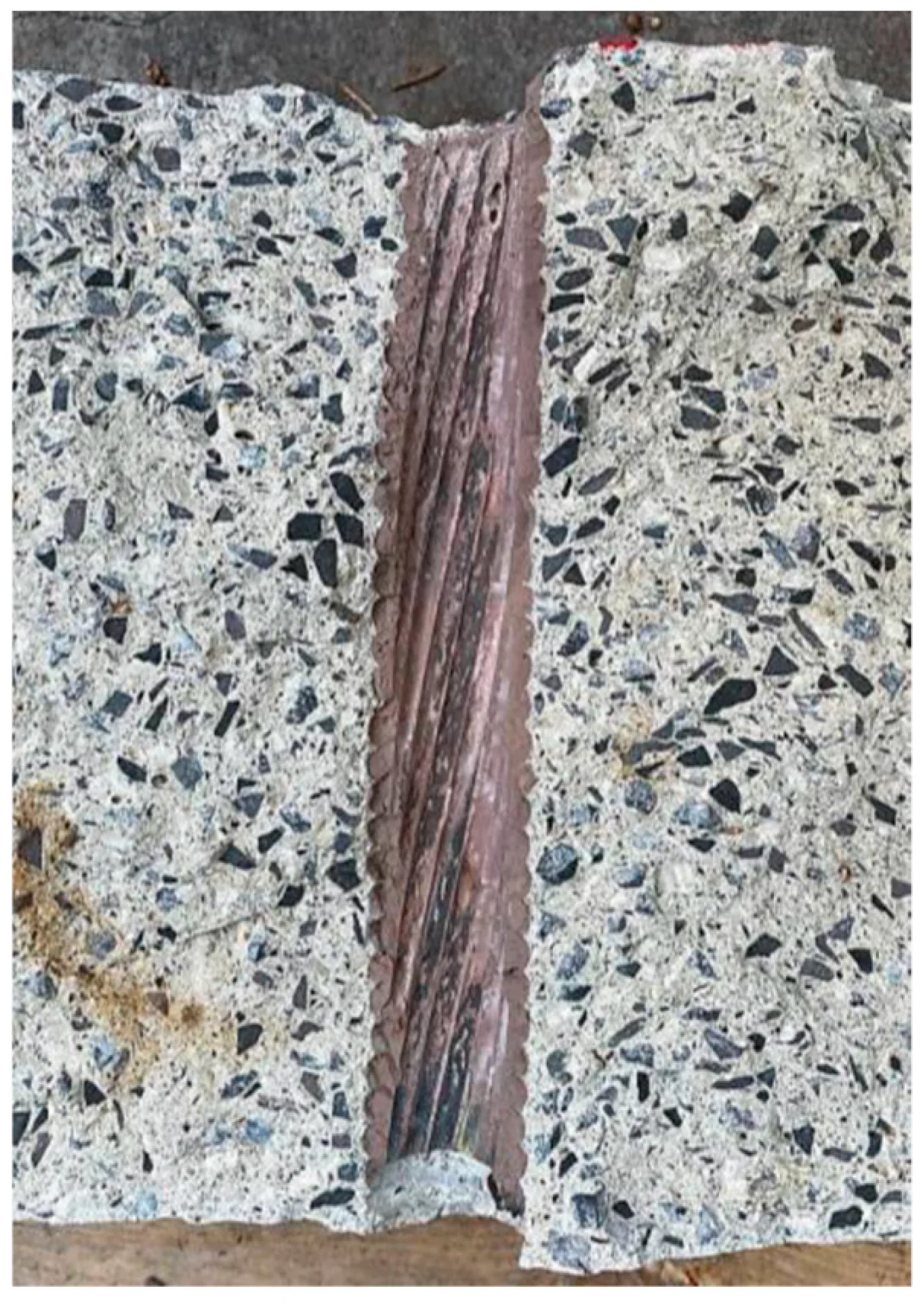
Interface failure between plain Sumo cable bolts and anchorage grout under the impact-loading condition [33].

Kong and Zhang [21] believed that when cable bolts whose diameter of 15.24 mm are subjected to impact loading, different failure modes may occur. They included slippage, rupture, and combined slippage and rupture. Under impact loading, for cable bolts whose diameter was 15.24 mm, the failure force was 260 kN. For these three different failure modes, their percentages were 14%, 37% and 49%. Wu et al. [22] conducted impact-loading tests on cable bolts with diameters of 17.8 mm and 21.6 mm. They also obtained three different failure modes, including slippage, rupture, and combined slippage and rupture. Additionally, they found that under impact loading, when the rupture of cable bolts occurred, the rupture position was around the locking plate. Zhang et al. [36] studied the failure modes of the cable bolts with constant resistance. Under multiple cyclic impact loading, the cable bolt will slip from the constant-resistance tube. Then, the cable-bolt rupture will not occur. Wu et al. [2] found that under impact loading, the wires of cable bolts became loose. During the loading process, the external steel wires are likely to rupture. The rupture position was commonly around the locking plate. Anzanpour et al. [33] studied the failure modes of the cable-bolt reinforcement under impact loading. Under the impact loading, bond failure suddenly occurred at the interface between the cable bolts and anchorage grout, as shown in Figure 6. However, due to the fact that the impact period was quite short, there was no apparent shear at the cable–grout interface. Instead, the cable bolts unscrewed from the anchorage grout. This can be experimentally confirmed by the fact that after the impact tests ended, there was no apparent failure of the rib geometry at the anchorage grout surface.

**Figure 7:**
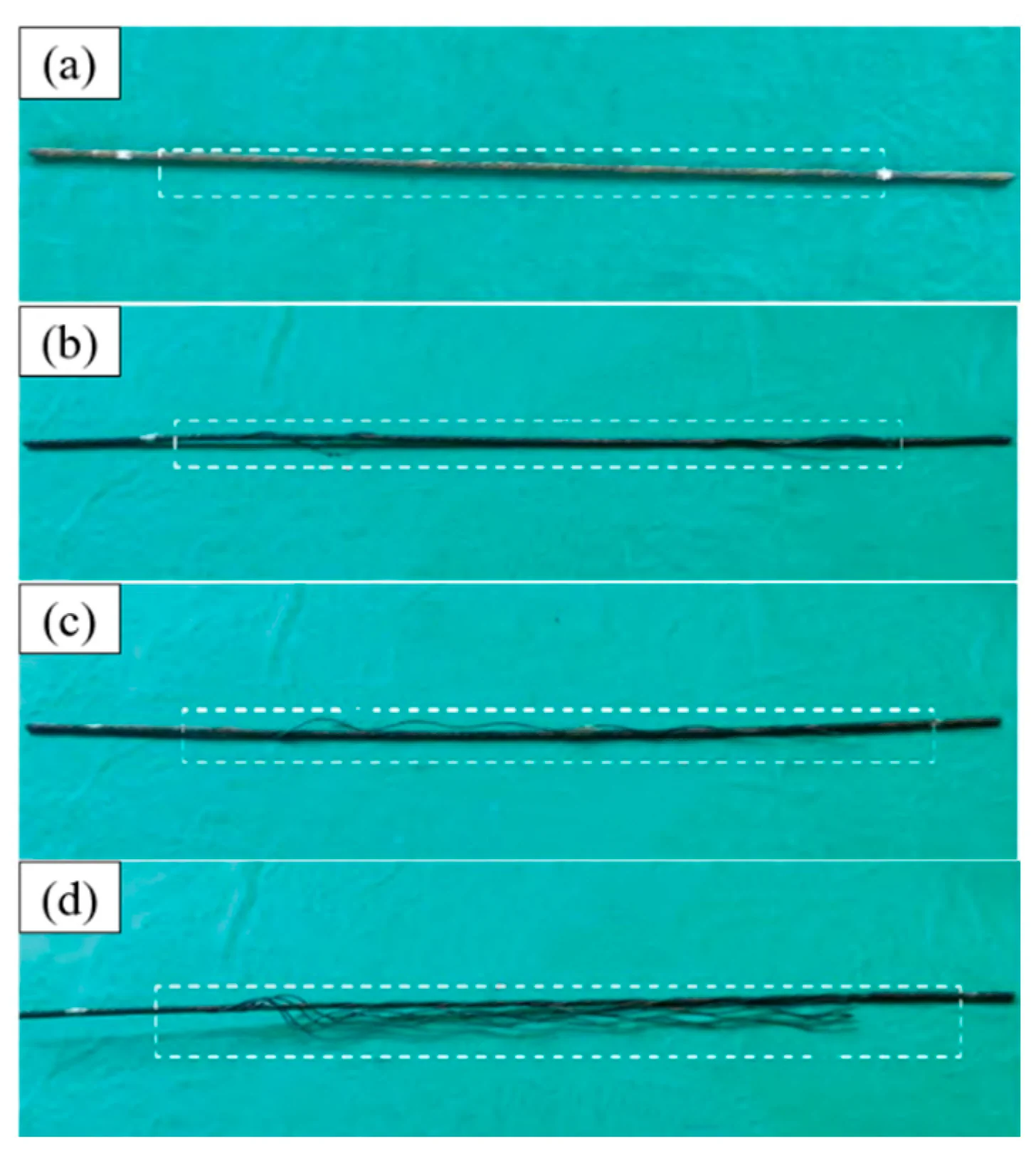
Failure modes of cable bolts under the impact loading with different energies: (**a**) 12,500 J; (**b**) 15,000 J; (**c**) 17,500 J; (**d**) 20,000 J [42].

Cheng et al. [42] found that after impact loading, the failure modes of the cable-bolt reinforcement were significantly related to the impact energy. When the impact energy was different, non-uniform deformation performance showed between steel wires of cable bolts. Specifically, the steel wires became loose, and the steel wires are likely to rupture. For example, when the cable bolt with a diameter of 17.8 mm was tested and the impact energy was less than 12,500 J, the cable bolts showed elastic deformation. When the impact energy reached 12,500 J, the cable bolt became loose and the steel wires did not rupture. When the impact energy ranged between 15,000 J and 17,500 J, a single steel wire of cable bolts will rupture, and the rupture position is located around the impact part. When the impact energy was around 17,500 J, apparent structural loosing will occur in cable bolts. When the impact energy ranged from 20,000 J to 22,500 J, more than 57% of steel wires in cable bolts will rupture. Then, the whole cable bolt lost its bearing capacity (Figure 7). Fu et al. [48] found that after traditional cable bolts were subjected to impact loading, brittle rupture failure of cable bolts will occur. Compared with that, when the cable bolts with high anti-impact toughness were subjected to impact loading, after rupture, the elongation rate was 1.52~1.82 times of traditional cable bolts. Therefore, under the impact loading, the steel wires in cable bolts with high anti-impact toughness are not likely to untwist.

## 5. Conclusions

(1) To evaluate the mechanical response and failure mechanisms of the cable-bolt reinforcement subjected to impact loading, two experimental tests methods are commonly used. One is the horizontal high-speed impact-loading method. The other one is the vertical drop-hammer impact-loading method. The shortcoming of the horizontal high-speed impact-loading method is that the applied impact energy is limited. Compared with that, when the vertical drop-hammer impact-loading method is used, the applied impact energy can be adjusted conveniently through changing the mass of the drop-hammer and the drop height.

(2) The cable-bolt structure plays a significant role in determining the mechanical response and failure status of the cable-bolt reinforcement subjected to impact loading. When traditional cable bolts are used, after the impact loading occurs, failure of the cable-bolt reinforcement may easily occur. Compared with that, modified cable bolts, such as the cable bolts with constant resistance and anti-impact rock (cable) bolts, can significantly sustain the impact energy. Additionally, the parameters including the impact energy and pretension force can also influence the mechanical response and failure modes of the cable-bolt reinforcement subjected to impact loading.

(3) The current study still has apparent shortcomings. Although the vertical drop-hammer impact-loading method is widely used, there are still several limitations. First, when this test is conducted, the air friction will lead to the result where a certain amount of energy is dissipated. However, during the test process, this dissipated energy is not considered. Second, during the impact-loading process, the hammer will generate certain elastic deformation. This elastic deformation will absorb certain energy. However, this absorbed energy is always neglected. Based on these shortcomings, further experimental impact-loading tests can be improved to evaluate the mechanical response of the cable-bolt reinforcement under the impact-loading condition.

## Figures and Tables

**Table 1:** Comparison between the impact-loading methods commonly used in the laboratory.

Test Method	Advantages	Disadvantages	Limitations
The horizontal high-speed impact-loading method	The cable bolt is placed along the horizontal direction. Therefore, it is convenient to place the cable bolt and adjust the configuration.	The impact velocity is relatively small. Consequently, the corresponding impact energy is limited.	For the case where high intensified impact energy needs to be simulated, the horizontal high-speed impact-loading method may not be appropriate.
The vertical drop-hammer impact-loading method	The impact velocity is relatively large. The impact energy can be adjusted conveniently via the drop-hammer mass and drop height.	The stability of the testing system decreased with the increasing of the overall height of the test rig. The hammer is located at the top. This requires experimenters to climb to the certain height to assemble and operate the test rig.	During the impact process, the weight of the drop-hammer is also applied on the cable bolts or plates. This may induce additional static loading force compared with the horizontal high-speed impact-loading method.

## Data Availability

No new data were created or analysed in this study. Data sharing is not applicable to this article.
